# Network analysis of cognitive function, glycemic–lipid profiles, and hepatic–renal function in individuals with diverse drinking patterns

**DOI:** 10.3389/fendo.2025.1553691

**Published:** 2025-07-30

**Authors:** Shuqi Xu, Ranran Zhao, Jincheng Wang, Xue Yang, Lan Wang, Cuixia An, Xueyi Wang, Ran Wang

**Affiliations:** ^1^ Department of Psychiatry, The First Hospital of Hebei Medical University, Shijiazhuang, Hebei, China; ^2^ Mental Health Center, Hebei Medical University, Shijiazhuang, Hebei, China; ^3^ Hebei Clinical Research Center of Mental Disorders, Institute of Mental Health, Shijiazhuang, Hebei, China; ^4^ Department of Psychiatry, Huzhou Third Municipal Hospital, The Affiliated Hospital of Huzhou University, Huzhou, Zhejiang, China

**Keywords:** primary funding cognitive function, glycemiclipid profiles, hepatic-renal function, drinking population, network analysis research data not shared

## Abstract

**Background:**

Harmful drinking habits can have a profound effect on individual health. However, there is currently a lack of network analysis studies on clinical indicators related to drinking population. The aim of this study was to investigate the relationships among drinking characteristics, cognitive functions, liver and kidney functions, and glucose and lipid levels in alcohol drinkers through the application of network analysis.

**Method:**

We conducted a stratified random sampling survey of 1,432 male employees in Gaocheng District, Hebei Province, in 2016. The Alcohol Dependence Scale (ADS) and the Alcohol Use Disorders Identification Test (AUDIT) were utilized to evaluate alcohol-related behaviors. Cognitive functions were assessed via the Hopkins Verbal Learning Test (HVLT), the Brief Visuospatial Memory Test (BVMT), Digit Symbol Coding Test (DSCT), and Digit Span Test (DST). Additionally, biochemical indicators such as blood glucose and lipid levels and hepatic and renal functions were measured. Analyses were performed to identify central symptoms and bridge symptoms of this network.

**Results:**

In our network analysis, the nodes representing TC, AST, AST/ALT, and ALT had the highest strength centrality. TC and AST presented the highest expected influence centrality. The closeness centrality indices for all the indicators performed well. The node DSCT ranked highly in terms of betweenness centrality.

**Conclusion:**

Correlations may exist among cognitive function, glycemic and lipid profiles, and hepatic–renal function in individuals with varying alcohol consumption patterns. Lipid and liver function indicators were identified as the most central factors in the network model. In the clinic, practitioners may focus on these abnormal central indicators as potential intervention targets to enhance the quality of life in alcohol drinkers.

## 1 Introduction

Alcohol is the most widely consumed addictive substance globally, with the World Health Organization (WHO) estimating that more than two billion people consume alcoholic beverages daily. Individuals over 15 years of age globally consume an average of 6.2 liters of pure alcohol per year (1), and excessive alcohol consumption can increase the risk of alcohol use disorders (AUDs). In recent years, the rate of high-risk drinking has significantly increased, with one in eight adults reporting such consumption within 12 months (2). Excessive drinking leads to a range of health, social, and behavioral problems, imposing a significant burden on families and societies.

Studies have shown that moderate alcohol consumption (up to 3 drinks/day) can reduce the risk of cognitive impairment by 30%, but it does not decrease the risk of cognitive decline (3). Compared to abstaining, drinking more alcohol was tied to a greater chance of abnormally rated hippocampal atrophy (4). Further findings revealed that heavier alcohol consumption (7 drinks per week) significantly lowers verbal fluency, although it does not markedly affect word memory or semantic fluency.

In addition to affecting cognitive functions, alcohol also significantly impacts liver and kidney functions as well as glucose and lipid levels. Evidence suggests that alcohol intake is positively correlated with biomarkers of liver damage, such as alanine aminotransferase (ALT), aspartate aminotransferase (AST), and γ-glutamyltransferase (GGT) (5). High-risk drinking behaviors can lead to alcohol-related liver damage, including alcoholic hepatitis, liver fibrosis, cirrhosis, and liver cancer (6), which are often the primary causes of alcohol-related deaths (7). Liver failure associated with drinking can result in hepatic encephalopathy, causing motor coordination and cognitive deficits (8). Chronic alcohol intoxication also affects renal filtration, leading to rapid deterioration of kidney function (9). Studies have revealed elevated levels of urea nitrogen, creatinine, and uric acid in heavy drinkers (10). Kidney dysfunction is linked to impairments in episodic memory, medial temporal lobe atrophy, and cortical thickness (11).

Cohort studies have confirmed that moderate drinking lowers the risk of type II diabetes, whereas high-risk drinking can increase the risk of metabolic syndrome (12, 13). Some research indicates that poor blood sugar control, including above-average levels and increased fluctuations, increases the risk of cognitive dysfunction (14, 15), although some longitudinal studies do not support this finding (16). Observational studies have demonstrated that excessive drinking causes abnormal lipid levels, with a dose–response relationship between alcohol intake and lipid levels, especially concerning high-density lipoprotein (HDL)-cholesterol (HDL-C), low-density lipoprotein (LDL)-cholesterol (LDL-C), and triglycerides (TGs) (17). In a 20-year follow-up study, individuals with higher lipid levels presented more significant cognitive decline than did those with healthy lipid levels (18). These dimensions are interconnected and influence each other, forming a complex clinical presentation, particularly in the central nervous system.

Although alcohol consumption directly and indirectly impacts brain function through the above multiple pathways, previous studies typically assessed this relationship via multivariate linear regression analysis after eliminating confounding factors (19, 20); however, evidence suggests that indicators influence each other, exhibiting complex interrelations (21). In the present study, we used network analysis, a novel visualization tool in which indicators serve as nodes and the relationships between them as edges, to construct a network for testing the above association. By calculating node and edge properties, among other network metrics, network analysis can be used to identify core influencing factors and bridging elements, accounting for partially controlled correlational links among conceptualized variables (22). Studies have shown that changes in the severity of certain indicators within the network can trigger changes in related symptoms (23), and interventions in core indicators can significantly affect the network structure, improving symptom prognosis (22).

Most prior empirical studies have used social network analysis to explore alcohol use among adults (24–27), focusing on how various characteristics of social network structures (e.g., homophily, popularity, transitivity) relate to individual drinking behaviors and exploring the impact of social mixing patterns on alcohol consumption. Moreover, some studies have utilized network analysis to investigate the relationships among lipoprotein profiles, cognitive functions, and symptoms of depression (28). However, there is currently a lack of network analysis research on the common clinical indicators among alcohol drinkers. Consequently, evidence for the relationship between the clinical characteristics of alcohol drinkers and alcohol consumption is limited.

The aim of this study was to investigate the relationships among drinking characteristics, cognitive functions, liver and kidney functions, and glucose and lipid levels in alcohol drinkers through the application of network analysis. This study’s results aid in revealing the pathophysiological mechanisms involving multiple systems in alcohol drinkers. Furthermore, identifying core and bridging factors within the network may help pinpoint key intervention directions for cognitive and physiological disorders in alcohol drinkers, with the aim of mitigating the adverse effects of unhealthy drinking patterns on cognitive and physiological health.

## 2 Methods

### 2.1 Participants

By employing stratified random sampling methods, a survey related to alcohol consumption was conducted from January to December 2016 among male employees in Gaocheng District, Shijiazhuang City, Hebei Province. First, the 13 towns in Gaocheng District were stratified by economic level, and four towns (Gangshang, Lianzhou, Qiutou, and Nanying) were randomly selected using simple random sampling via a random number table. Subsequently, factories in these towns were stratified by size, from which 10 factories were randomly selected using simple random sampling. Finally, 1,539 male employees were randomly surveyed with stratification by job position. After 107 individuals with incomplete information were excluded, 1432 participants were deemed eligible for this study. The inclusion criteria were as follows: (1) male employees in active service; (2) aged over 18 years; (3) had a history of alcohol consumption; and (4) had undergone relevant physical and laboratory examinations. The exclusion criteria were inability to understand the content of the questionnaire and inability to participate and complete the survey and evaluation. The participants were informed of the specific details of the study prior to their involvement and, upon mutual agreement, signed written informed consent forms. This research project was approved by the Ethics Committee of the First Hospital of Hebei Medical University (No. 2015042) and clinical trial registration number is ChiCTR-ICC-15007244.

### 2.2 Measures

In this study, participants underwent assessments via structured questionnaires to gather comprehensive data on sociodemographic characteristics. The Alcohol Dependence Scale (ADS) (29) was used to evaluate the degree of individual alcohol dependence. The Alcohol Use Disorders Identification Test (AUDIT) (30), recommended by the WHO, was employed to assess the drinking behavior of participants in the past year, categorizing them into high-risk (AUDIT ≥ 8) and low-risk (AUDIT < 8) drinking groups on the basis of a threshold of 8 points. The assessments were conducted by psychiatrists from the First Hospital of Hebei Medical University, with all the evaluators passing a consistency assessment test, resulting in an interrater reliability coefficient of 0.87.

Various scales were employed for a comprehensive evaluation of the cognitive functions of the participants: the Hopkins Verbal Learning Test (HVLT) (31), which includes total and delayed scores, assesses verbal learning and memory abilities, and provides insights into participants’ performance in vocabulary learning, immediate memory, delayed memory, and recognition memory. The Brief Visuospatial Memory Test (BVMT) (32), which includes total and delayed scores, was used to measure visuospatial memory function and reflects participants’ ability to encode, store, and recall visual information. The Digit Symbol Coding Test (DSCT) (33), a psychological test assessing processing speed and working memory, involves the pairing of numbers with corresponding symbols within a designated time, which indicates participants’ information processing speed and attention. The Digit Span Test (DST) (34), which is primarily used to evaluate short-term and working memory abilities, includes forward (DST-F) and backward (DST-B) tasks and involves the assessment of participants’ memory storage and manipulation abilities through the recall of number sequences. Biochemical indicators for glycemic and lipid levels included fasting blood glucose (GLU), total cholesterol (TC), TG, HDL, and LDL levels. The hepatorenal function indicators included ALT, AST, the AST/ALT ratio, GGT, blood urea nitrogen (BUN), creatinine (Cr), and uric acid (UA).

### 2.3 Data analysis

#### 2.3.1 Network estimation

We constructed a network model using data from 1,432 participants and carried out network analysis employing several R packages (35), including “bootnet,” (36) “dplyr,” (37)”magrittr,” (38)”psych,” (39)and “qgraph. “ (40) The network was estimated via the extended Bayesian information criterion (EBIC) Glasso method and Spearman correlation analysis (41). Visualization of the network was performed via the Fruchterman-Reingold algorithm (42), which positions nodes with more connections centrally within the network, arranges nodes closer to each other on the basis of stronger interconnections, and depicts stronger correlations with thicker edges. Edge stability within the network was assessed via the bootnet function with 1,000 bootstrap samples (43). Furthermore, node stability was evaluated by calculating the correlation stability coefficient (CS-C) through a case-dropping bootstrap approach with 2,500 samples, whereby stability was measured by systematically removing samples (44). A CS-C value exceeding 0.25 suggests moderate stability, whereas a value above 0.5 indicates strong stability (43).

#### 2.3.2 Network properties

In our study, we utilized node metrics to assess the importance and potential clinical associations of each node. This approach incorporates several common centrality indices: *strength*, *closeness*, *betweenness*, and *expected influence*. These indices enabled us to quantify the relationships and network structure related to participants’ alcohol consumption characteristics, glycolipid levels, and hepatic and renal functions, as well as their cognitive abilities. *Strength* refers to the sum of the weights of edges connected to a node. A higher strength value suggests that the node has stronger or more numerous connections, potentially influencing a greater number of neighbors, thereby indicating its importance. *Closeness centrality* measures the proximity of a node to all other nodes in the network. It is calculated as the inverse of the sum of the shortest path lengths from the node to all others, implying that nodes with higher closeness centrality can interact or communicate more efficiently with others, signifying a more central position in the network. *Betweenness centrality* quantifies the frequency with which a node acts as a bridge along the shortest path between two other nodes. This reflects the degree to which a node lies on paths connecting other network components. Nodes with high betweenness can significantly influence the flow of information within the network, as they link different parts of it. *Expected influence* accounts for both the strength and direction of connections associated with a node, predicting the extent of a node’s projected impact on others.

#### 2.3.3 Network comparison

We employed the “NetworkComparisonTest” package in R to analyze the network differences between the high-risk drinking (HRD) group and the low-risk drinking (LRD) group. This analysis, which was executed with a total of 1000 iterations, facilitated a comprehensive comparison of network structures and strengths among individuals grouped by different levels of drinking risk, providing insights into the network invariance and global strength invariance between the two groups.

## 3 Results

### 3.1 Participant characteristics

In the present study, we examined a cohort of 1,539 participants. Some participants were excluded due to incomplete data from specific scales; therefore, a total of 1,432 individuals were incorporated into the network analysis. Among them, 828 (57.82%) were categorized as low-risk drinkers, whereas 604 (42.18%) were identified as high-risk drinkers. The demographic and clinical characteristics of the study population are presented in **Table 1**. One-way ANOVA revealed that there were no significant differences in these characteristics among the three groups (p>0.05).

**Table 1:** Demographic and clinical characteristics of different alcohol consumption risk groups.

Indices	Total (n=1432)	Low-risk drinking group (n=828)	High-risk drinking group (n=604)
Age, year	36.67 ± 10.36	35.81 ± 10.11	37.86 ± 10.59
Education, year	3.75 ± 0.99	3.72 ± 0.97	3.79 ± 1.01
Cognitive Function			
HVLT-Total	23.63 ± 6.68	23.86 ± 5.72	23.42 ± 5.26
HVLT-Delay	8.56 ± 2.46	8.68 ± 2.42	8.47 ± 2.47
BVMT-Total	25.59 ± 8.86	25.65 ± 7.62	25.29 ± 7.37
BVMT-Delay	10.25 ± 2.51	10.33 ± 2.48	10.22 ± 2.55
DSCT	53.97 ± 15.35	54.45 ± 14.45	53.26 ± 15.44
DST-F	7.62 ± 6.70	7.14 ± 1.45	7.09 ± 1.49
DST-B	5.51 ± 6.84	5.00 ± 1.52	5.07 ± 1.39
Alcohol Consumption			
AUDIT	6.97 ± 5.59	3.1 ± 2.48	11.88 ± 3.48
ADS	2.80 ± 3.68	1.26 ± 2.02	4.56 ± 4.11
Glycolipid Levels			
GLU, mmol/L	5.35 ± 1.23	5.30 ± 1.32	5.40 ± 1.06
TC, mmol/L	4.65 ± 0.99	4.55 ± 0.96	4.79 ± 1.03
TG, mmol/L	1.67 ± 4.10	1.47 ± 1.65	2.00 ± 6.36
HDL, mmol/L	1.24 ± 0.32	1.20 ± 0.27	1.29 ± 0.38
LDL, mmol/L	2.95 ± 0.81	2.92 ± 0.78	3.00 ± 0.86
Hepatorenal Function			
ALT, U/L	29.15 ± 20.34	28.32 ± 19.38	30.53 ± 21.64
AST, U/L	24.32 ± 10.02	23.79 ± 8.56	25.08 ± 11.97
AST/ALT	1.01 ± 0.42	1.02 ± 0.45	0.97 ± 0.37
GGT, U/L	33.53 ± 31.12	27.63 ± 21.76	42.32 ± 38.71
BUN, mmol/L	5.23 ± 10.17	4.96 ± 1.18	5.69 ± 16.55
Cr, μmol/L	76.22 ± 11.83	76.76 ± 11.64	75.49 ± 11.91
UA, μmol/L	347.96 ± 88.79	340.84 ± 85.64	356.93 ± 93.04

Data are mean ± SD. Some participants had missing data for related items. HVLT, Hopkins verbal learning test; BVMT, brief visuospatial memory test; DSCT, digit symbol coding test; DST-F, digit span test in the forward condition; DST-B, digit span test in the backward condition; AUDIT, alcohol use disorders identification test; ADS, alcohol dependence scale; GLU, serum glucose; TC, total cholesterol; TG, triglyceride; HDL, high-density lipoprotein; LDL, low-density lipoprotein; ALT, alanine aminotransferase; AST, aspartate aminotransferase; GGT, γ-glutamyl transferase; BUN, blood urea nitrogen; Cr, creatinine; UA, uric acid.

### 3.2 Correlations of cognitive function, glycemic–lipid profiles and hepatic–renal function

The strong correlations (i.e. *r* > 0.7) were found between AST and ALT, LDL and TC, and GGT and ALT as we expected. The moderate correlation (i.e. 0.3< *r <*0.7) was seen in TG and TC, TC and GGT, and AST and GGT. However, no correlation was found in the cognitive function indicators. The detailed results are shown in **Supplementary Figure 1**.

### 3.3 Network structure

**Figure 1:**
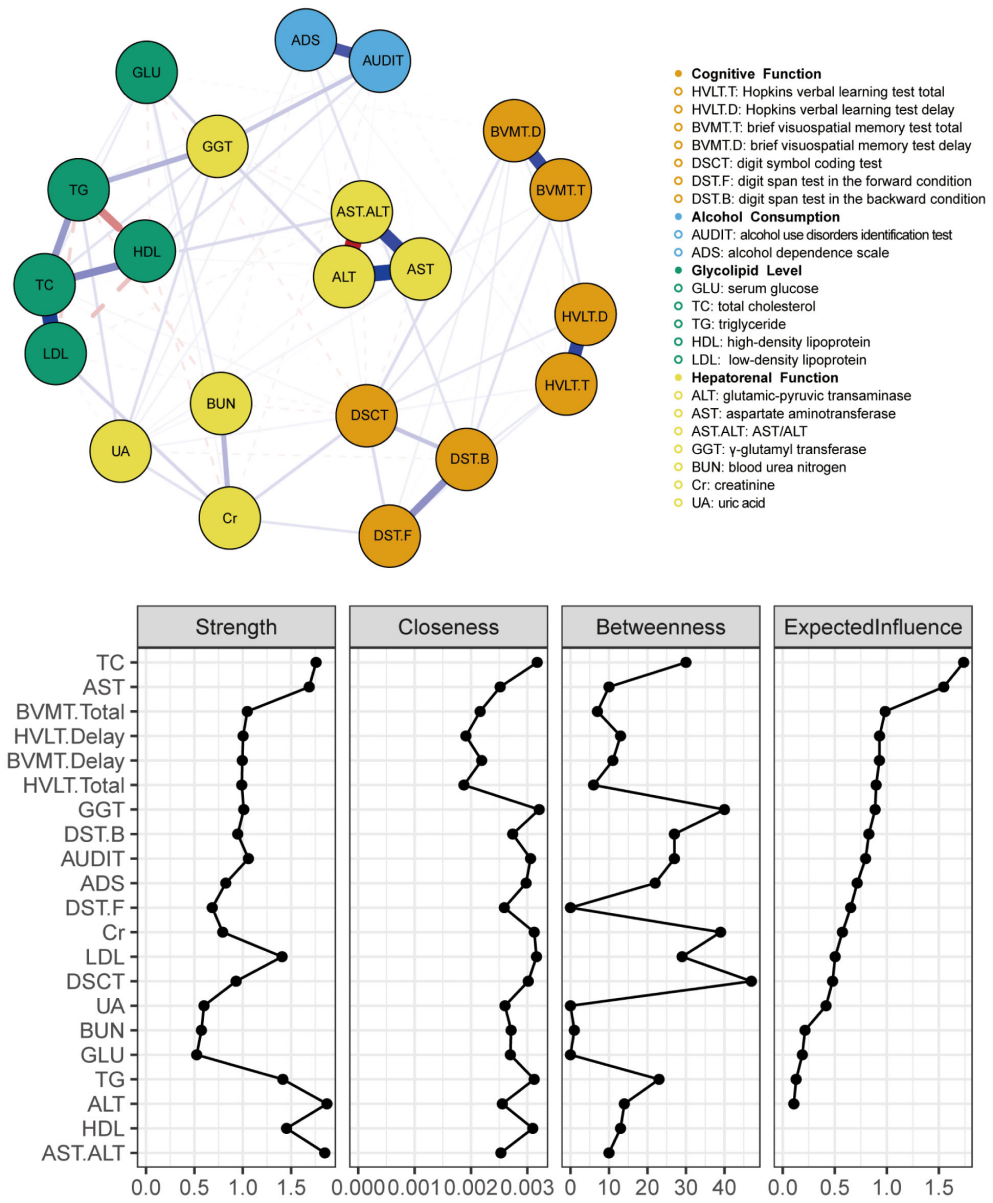
Clinical characteristics, indicator network, and centrality measures of the whole study sample (N = 1432).

The network diagram in **Figure 1** illustrates the correlations among participants’ cognitive functions, alcohol consumption status, glucose–lipid levels, and hepatic–renal functions. In the diagram, each circle symbolizes a named node within the network. The connecting lines indicate the intensity of the relationships between these nodes. The nodes AST/ALT and ALT had the strongest connections.

In the network characterizing the features of individuals who consume alcohol, measures of centrality delineate the strength, closeness, betweenness, and expected influence value of each node. Elevated values suggest a more central position of the item within the network. The TC, AST, AST/ALT, and ALT nodes presented the highest strength in centrality indices. This signifies that the total weights of the links directed toward these four nodes were predominant. The GGT node exhibited the greatest closeness, indicating that this liver function parameter was instrumental in linking other symptoms that lacked direct connections in the network. Among the betweenness centrality indices, the DSCT, GGT, and Cr nodes were the most prominent. These three nodes, which served as significant mediators, acted as bridges linking other symptoms. The TC and AST nodes presented the greatest expected influence, suggesting their strong positive relationships with other nodes.

As shown in **Figure 1**, the ALT (strength = 1.870; betweenness = 14) and the AST/ALT ratio (strength = 1.853; betweenness = 10) nodes presented the highest strength values. Additionally, TC (strength = 1.758; betweenness = 30) and AST (strength = 1.687; betweenness = 10) also demonstrated elevated strength parameters. Notably, these four nodes were identified as having the highest nodal strength in the network. However, regarding the expected influence, only the indices for TC (1.738) and AST (1.546) showed a significant predominance over the other metrics. Intriguingly, the expected influence values for HDL and the AST/ALT ratio were absent. Considering that expected influence usually denotes a node’s prospective impact within a network, this absence might indicate a relative lack of influence of these two nodes within the network context.

### 3.4 Network stability analysis

**Figure 2:**
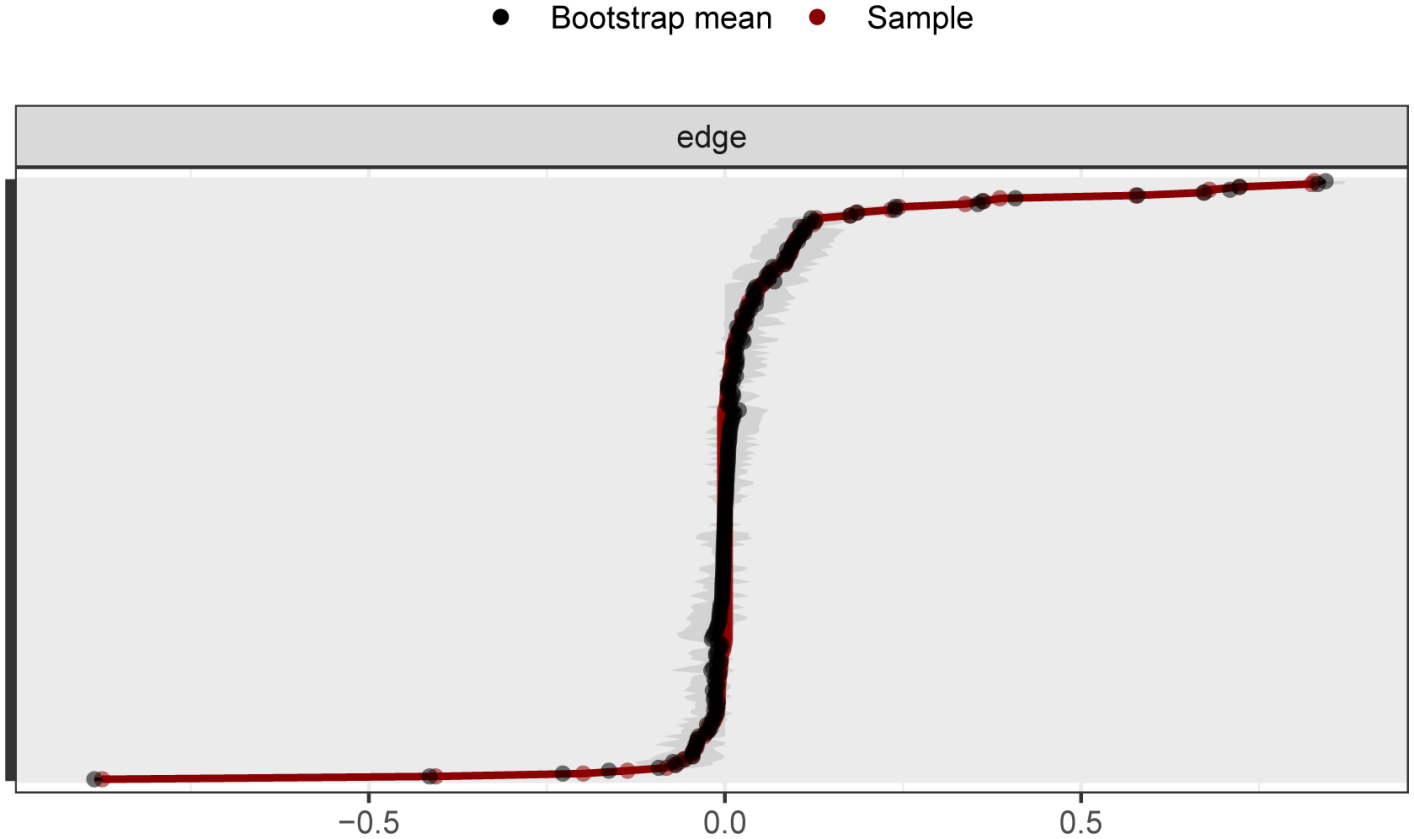
95% confidence intervals for edge weights in network analysis (bootstrap method). The red lines indicate the edge weight values, the black lines represent the average edge weights under the bootstrap method, and the gray areas depict the 95% confidence intervals for the edge weights.

**Figure 3:**
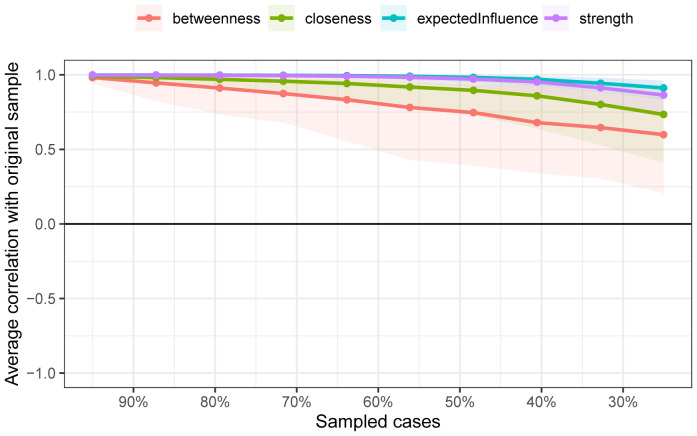
Node stability outcomes in network analysis. The horizontal axis represents the proportion of the sample included, whereas the vertical axis indicates the correlation between the centrality measures of nodes in the original network and those in the network with a proportionally included sample.

As shown in **Figure 2**, the bootstrap results demonstrated that the 95% confidence intervals for the edge weights are relatively narrow, indicating high edge stability in the network. As depicted in **Figure 3**, the application of the bootnet function with a case-dropping bootstrap method revealed that the CS-C values are all above 0.5, suggesting excellent node stability in the network.

### 3.5 Comparison of networks between the HRD and LRD groups

Our analysis did not reveal significant differences in the overall structure or global strength between the HDR and LDR networks (see **Supplementary Figure 2**). The results of the network invariance test and the global strength invariance test indicated that there were no significant differences in the overall structure (*M*=0.532, *p*=0.123) or global strength (*S*=2.679, *p*=0.381) between the two groups of networks.

## 4 Discussion

The purpose of this study was to investigate the relationships among the clinical characteristics, laboratory parameters, and cognitive function of individuals who consumed alcohol. We recruited 600–800 age-matched (mainly middle-aged, i.e., 30–40 years old) volunteers. We conducted a Spearman correlation analysis, and the results showed that there was no significant correlation between physiological indicators and cognitive function. However, when we integrated the various indicators into the network analysis, we found some noteworthy results.

In our network analysis, the variability in node strength centrality for the 21 indicators in alcohol drinkers was estimated. “Strength” denotes the extent of interaction or connection between nodes, providing insights into a node’s activity within the network or its interaction intensity with other nodes. The nodes representing TC, AST, AST/ALT, and ALT had the highest strength centrality, indicating that these indicators are more closely connected with other health parameters and play a key role in the overall symptom network of alcohol drinkers. “Expected influence” quantifies a node’s anticipated impact within the network. A high expected influence of a node means that it affects not only its direct neighbors but also the neighbors of its neighbors. Our results indicate that TC and AST exhibited the highest expected influence centrality, suggesting that interventions targeting lipid levels and liver function may alleviate other symptoms within the glucose–lipid–hepatic–renal–cognitive symptom network in alcohol drinkers (45). However, the linkages of these central nodes with the cognitive domain were significant but more modest than we expected. This might be because we oversaw the cumulative effects of alcohol consumption over time as well as the types of alcohol consumed (i.e., alcohol concentrations). In addition, some studies suggest that high centrality may indicate that a symptom is the “causal endpoint for many pathways in the data” (46) rather than the starting point, implying that targeting that symptom may not necessarily improve the entire network (46, 47). Thus, causal relationships among other indicators in the network should be considered comprehensively (46). With respect to these indices, future studies may be warranted to clarify the impact of daily alcohol consumption on cognitive functions.

The closeness centrality indices of all nodes showed consistent patterns, as shown in **Figure 1**. “Closeness centrality” offers positional information within the network; a node with higher closeness suggests that it is, on average, less distant from other nodes, implying ease in spreading information or being influenced by others within the network.

Notably, the node representing the cognitive function—DSCT—ranked highly in terms of betweenness centrality. Nodes with high “betweenness centrality” act as bridges within the network, as many information paths pass through these nodes. They hold significant control within the network by frequently connecting different parts or communities. The results indicate that DSCT acts as a “bridge” in the network, playing a key role in transmitting information or signals. A previous meta-analysis showed that DSCT is more sensitive for detecting processing speed differences in subjects than other cognitive domains, such as working memory and attention (48). Impaired DSCT is also associated with cognitive dysfunction in drinking populations (49). A longitudinal study showed that better processing speed-related neurocognitive function increases the likelihood of alcohol-dependent patients managing their addiction through exercise coping strategies (50), whereas those with poorer cognitive function require more complex training approaches, such as multimodal and interactive methods. Therefore, in addition to directly targeting central indicators for intervention, employing appropriate coping strategy training may be a key approach to improving the overall health of individuals who consume alcohol.

This study has several limitations that need to be acknowledged. First, the use of cross-sectional data limits our ability to infer the directionality of relationships between health indicators in alcohol drinkers. Future research could employ longitudinal designs to explore these candidate indicators and help assess potential causal relationships, ultimately aiding the development of more effective interventions. Second, our sample comprised individuals with varying drinking patterns rather than a homogenous samples of individuals with pathological drinking behaviors, limiting the generalizability of the findings to AUD patients at different clinical stages. Future studies should include larger-scale investigations. Third, certain factors associated with cognitive function in drinking populations were not included in the network analysis. Future research should incorporate additional neuropsychological variables, such as anxiety, depression, and social functioning, for further analysis.

In conclusion, there may be significant but modest correlations among cognitive function, glycemic and lipid profiles, and hepatic–renal function in individuals with varying alcohol consumption patterns. Lipid and liver function indicators were identified as the most central factors in the network model. Both excessive and moderate alcohol consumption can elevate these core biomarkers.These central indicators may indirectly activate other factors within the network, creating a self-reinforcing feedback loop (51, 52). Targeting key symptoms may influence this cycle and presumably affect the overall disease state (53). Therefore, clinical practice may focus on these abnormal central indicators as potential intervention targets to increase the quality of life in alcohol drinkers.However, current evidence suggests that alcohol avoidance may be the only risk-free option.

## Data Availability

The raw data supporting the conclusions of this article will be made available by the authors, without undue reservation.
