# Supplementary material for: Network analysis of cognitive function, glycemic–lipid profiles, and hepatic–renal function in individuals with diverse drinking patterns
Source: Front Endocrinol (Lausanne). 2025 Jul 30;16:1553691. doi: 10.3389/fendo.2025.1553691 (PMC12343226; doi:10.3389/fendo.2025.1553691)
Supplement: Supplementary file 1 [file DataSheet1.docx]

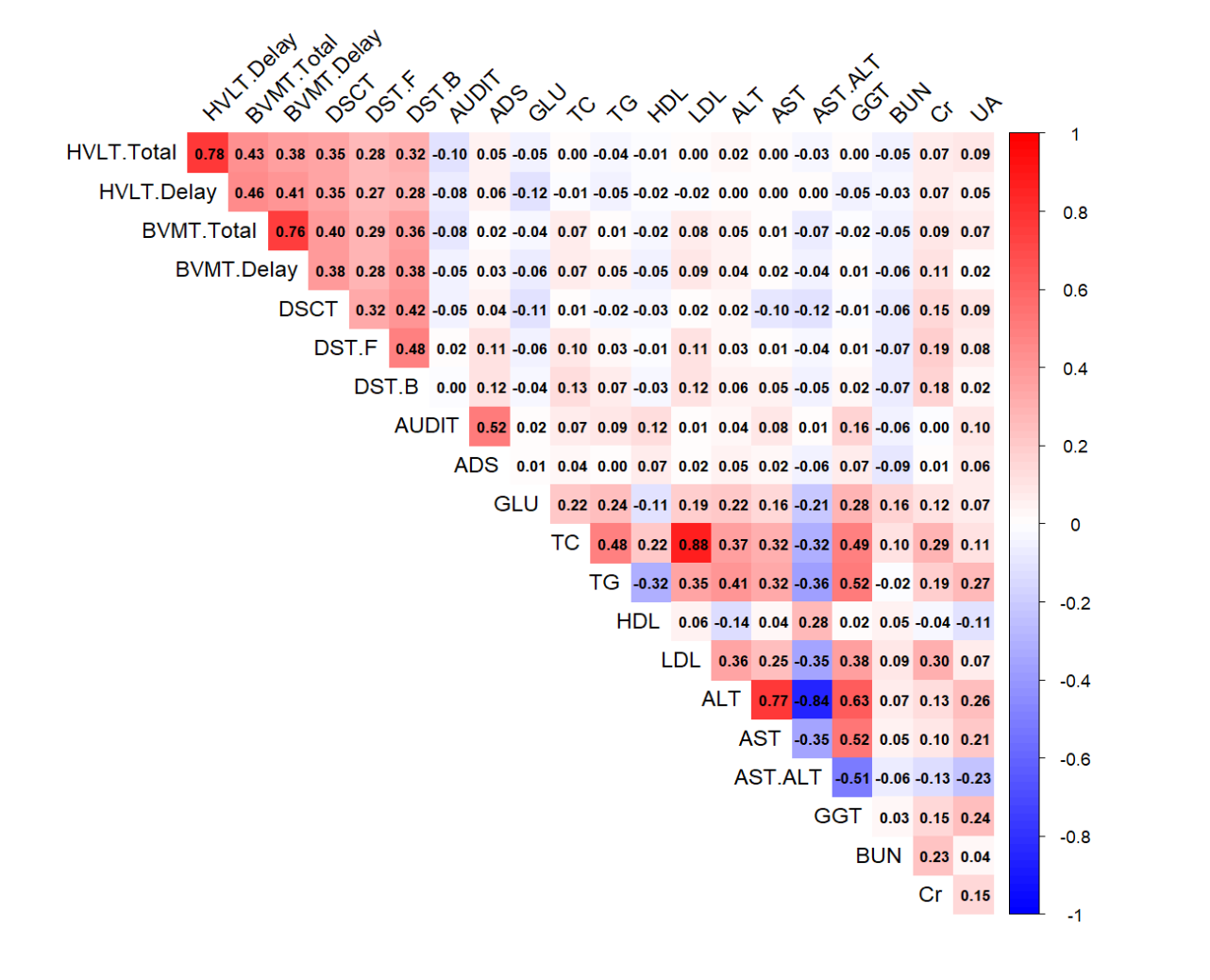
**Supplementary Figure 1.** Correlation matrices of cognitive function, glycemic lipid profiles and hepatic-renal function. Blue signifies a negative correlation, while red denotes a positive correlation. The intensity of the color indicates the strength of the correlation.

**Supplementary Figure
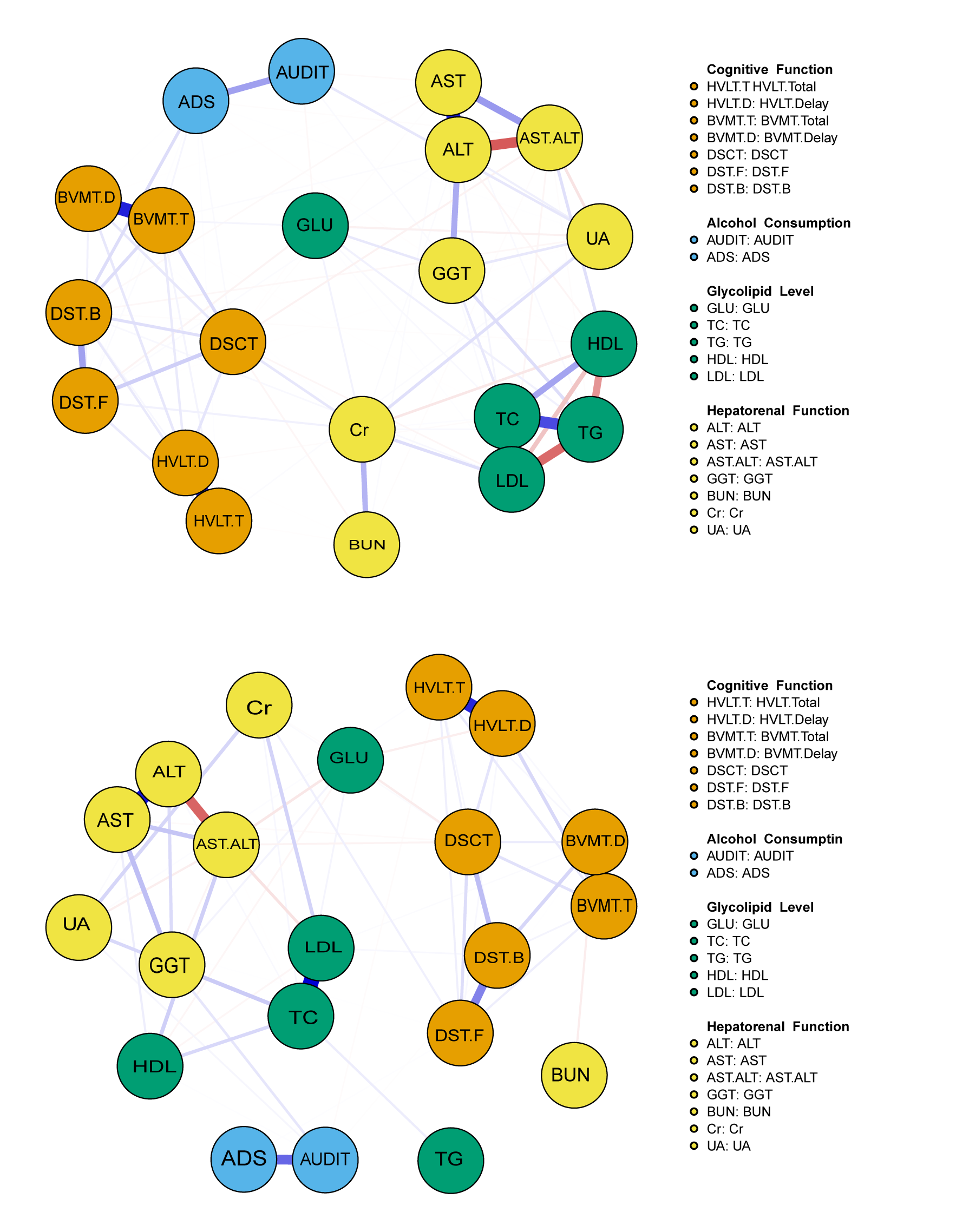
2.** Comparison of networks between the high-risk drinking and low-risk drinking groups.
